# Capability of airline jets as an observation platform for noctilucent clouds at middle latitudes

**DOI:** 10.1186/s40645-022-00469-4

**Published:** 2022-01-29

**Authors:** Hidehiko Suzuki, Ayako Matsumoto, Peter Dalin, Yuriko Nakamura, Satoshi Ishii, Kazuyo Sakanoi, Kaori Sakaguchi, Taku Takada, Takuo T. Tsuda, Yuta Hozumi

**Affiliations:** 1 Meiji University, Kawasaki, Kanagawa Japan; 2 ANA HOLDINGS INC., Minato-ku, Tokyo Japan; 3 Swedish Institute of Space Physics, Kiruna, Sweden; 4 Komazawa University, Setagaya, Tokyo Japan; 5 National Institute of Information and Communications Technology, Koganei, Tokyo Japan; 6 Tokyo Metropolitan College of Industrial Technology, Arakawa, Tokyo Japan; 7 The University of Electro-Communications, Chofu, Tokyo Japan

**Keywords:** Noctilucent cloud, Mesosphere, Middle latitudes, Global warming, Airline, Aircraft

## Abstract

The exact occurrence frequency of noctilucent clouds (NLCs) in middle latitudes is significant information because it is thought to be sensitive to long-term atmospheric change. We conducted NLC observation from airline jets in the Northern Hemisphere during the summer 2019 to evaluate the effectiveness of NLC observation from airborne platforms. By cooperating with the Japanese airline All Nippon Airways (ANA), imaging observations of NLCs were conducted on 13 flights from Jun 8 to Jul 12. As a result of careful analysis, 8 of these 13 flights were found to successfully detect NLCs from middle latitudes (lower than 55° N) during their cruising phase. Based on the results of these test observations, it is shown that an airline jet is a powerful tool to continuously monitor the occurrence frequency of NLCs at midlatitudes which is generally difficult with a polar orbiting satellite due to sparse sampling in both temporal and spatial domain. The advantages and merits of NLC observation from jets over satellite observation from a point of view of imaging geometry are also presented.

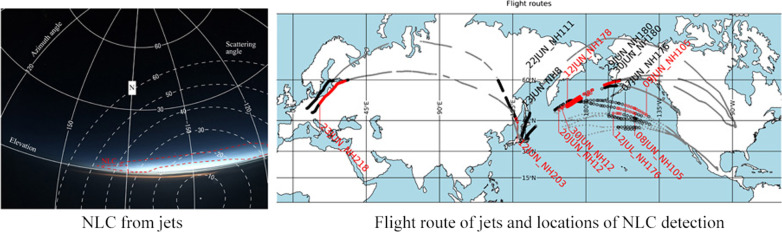

## Introduction

Noctilucent clouds (NLCs) are the Earth’s highest clouds, and they appear at the mesopause region during summer in both subpolar regions (Gadsden and Schröder 1989). NLCs are a proxy for monitoring climate change because their occurrence is highly sensitive to the mixing ratio of water vapor and atmospheric temperature in the mesopause region (Thomas 1996; Thomas and Olivero 2001). Several models predict that global warming in the lower atmosphere caused by global increases of greenhouse gasses promotes cooling of the upper atmosphere (e.g., Lübken et al. 2013). In addition, photochemical reactions involving greenhouse gasses, such as methane, increase the mixing ratio of water vapor in the mesosphere, which is a source of water ice clouds (i.e., NLCs) (Hervig et al. 2016). Thus, enhancement of global warming due to greenhouse gasses can extend the NLC region toward latitudes lower than the current typical subpolar latitudes (Thomas 1996). Therefore, it is important to know the occurrence of NLCs in middle latitudes precisely. A Japanese research group started NLC observation from the ground in northern Japan in 2012. They have reported the first detection of NLCs from multiple sites in Hokkaido, Japan (43.17° N–45.36° N), on June 21, 2015 (Suzuki et al. 2016). However, they have not reported further NLC events from Japan from 2016 to 2018 although radar observations sometimes detect mesospheric echoes. One possible cause that prevents the detection of visible NLCs from the ground is poor weather conditions during the early summer season in Japan (Suzuki et al. 2016). Satellite observations are a powerful tool to investigate the global distribution and a long term variability of NLC (or Polar Mesospheric Clouds, PMCs, as observed from space). In 2007, NASA launched the Aeronomy of Ice in the Mesosphere (AIM) satellite to monitor the PMCs (Russell et al. 2009; Lumpe et al. 2013). The AIM satellite, which is still in operation, provides data regarding the temporal and spatial variations of PMCs over both polar regions. However, data availability in middle latitudes is quite limited due to an observation geometry and a sparse sampling caused by a polar orbit. Recently, the observation of PMCs from Geostationary Earth Orbit (GEO) meteorological satellite is reported (Tsuda et al. 2018). This method is quite effective to continuously monitor the PMCs in both hemispheres. Hozumi et al. (2021) performed derivation of horizontal winds at altitudes of PMCs by tracking a motion of the PMCs observed by Japanese GEO satellite, Himawari-8. However, GEO satellite can monitor the PMCs in fixed latitude and longitudinal ranges. Therefore, we have started a feasibility study to monitor NLCs in middle latitudes from airplanes and balloons in addition to current ground-based imagers since June 2019. International jets fly at an altitude of about 10 km during the cruising phase. From this altitude, NLC observation is possible without obstruction by lower clouds because most tropospheric clouds are below this altitude. Our project has collaborated with the Japanese airline All Nippon Airways (ANA). This paper reports the results of NLC observations in the middle latitude region using airline jets. An initial result of NLC observations with small cameras installed in the cockpit of passenger jets connecting North America, Europe, and Japan is reported in detail. The instrumental overview, details of the observation plan, and the analysis method are described in Sect. 2. Results of NLC observations by airline jets conducted in the Northern Hemisphere during the summer 2019 are shown in Sect. 3. Occurrence of NLCs in middle latitudes and the advantages of observation with jets are discussed in Sect. 4.

## Instrumentation and analysis method

We entrusted small action cameras (GoPro HERO7 Black) to ANA and conducted test observation of NLCs from cockpits in the northern summer season of 2019. We also tested a GoPro camera to check its sensitivity by taking star images in a dark sky. It was found that using the “nightlapse mode,” the GoPro can capture star field images. If an exposure time and sensitivity are set to 30 s and ISO 800, respectively, Milky way can be detected (It means GoPro can capture faint stars with visual magnitude of up to 5–6). Because NLCs are brighter than visible stars, the GoPro HERO 7 Black was confirmed to be sensitive enough to detect NLCs. The shooting interval was set to 1 min during each flight except for a flight on Jul 12 (see below for details).

Several ANA flights from Japan to Europe and the USA cruise subpolar regions. We checked which flight routes are suitable for NLC observation during the summer solstice before an observation planning. The criteria were (1) the flight route includes a latitude higher than 40° N and (2) the solar elevation was between − 15° and − 5° during the cruising in a subpolar region. Table 1 lists the ANA flights connecting Japan–Europe and Japan–North America that satisfy these criteria. These flights are nominated by considering their typical route provided by Flightradar24, which is a web service providing real-time and past commercial aircraft flight tracking information (https://www.flightradar24.com/). A position of the Sun in celestial coordinates is assumed to be that on the summer solstice for the calculation of a local solar elevation angle during a flight. These flights are regular routes which are nominally operated every day. We have conducted test observations with some of these flights on several days. The dates on which the NLC observations were conducted are also listed. Underscored dates mean that NLCs were successfully detected on that date. Dates with an asterisk mean that NLCs were detected from a latitude lower than 55°N. It is noted that the observation on Jul 12 during flight NH176 was conducted with another compact digital camera (Canon PowerShot G7 X Mark II) belonging to a crewmember of the flight. In total, NLC imaging observations were conducted on 13 flights in the Northern Hemisphere during the summer of 2019. NLCs were detected from middle latitudes (lower than 55°N) on eight flights of these flights.

**Table 1:** List of flights operated by ANA considered to be suitable for NLC observation

Flight ID	Departure	Destination	Total flight time (h)	Flight time satisfying the criteria (h)	Observation date Day/Month (of 2019)
*Japan–Europe flights*					
NH203	Tokyo	Frankfurt	11.7	1.7	12/Jun* (Fig. 3d)
NH218	Munich	Tokyo	10.9	1.9	23/Jun*(Fig. 3f)
NH206	Wien	Tokyo	10.4	2.9	Not tested
*Japan–America flights*					
NH6	San Francisco	Los Angeles	9.8	1.3	Not tested
NH106	Tokyo	Los Angeles	9.8	0.5	Not tested
NH105	Los Angeles	Tokyo	11.1	5.0	08/Jun*(Fig. 3a), 09/Jun*(Fig. 3b)
NH8	Tokyo	San Francisco	9.3	1.3	13/Jun
NH172	Tokyo	San Jose	9.3	0.9	Not tested
NH178	Tokyo	Seattle	8.8	0.8	12/Jun*(Fig. 3c)
NH109	New York	Tokyo	13.2	0.5	Not tested
NH174	Tokyo	Houston	12.0	1.4	Not tested
NH12	Tokyo	Chicago	11.8	3.1	20/Jun*(Fig. 3e), 30/Jun*(Fig. 3g)
NH112	Tokyo	Chicago	11.8	1.8	Not tested
NH111	Chicago	Tokyo	12.3	0.1	22/Jun
NH116	Tokyo	Vancouver	8.7	0.9	Not tested
NH180	Tokyo	Mexico	11.4	1.0	29/Jun, 30/Jun
NH176	Tokyo	Los Angeles	9.8	1.3	07 Jun, 12/Jul*(Fig. 3h)

Duration time satisfying the criteria for the NLC observation (see text) is also shown. The dates on which NLC observations were conducted are also listed. Underscored dates denote successful NLC detection on that date. Dates with an asterisk mean that NLCs were detected at a latitude lower than 55°N

**Fig. 1:**
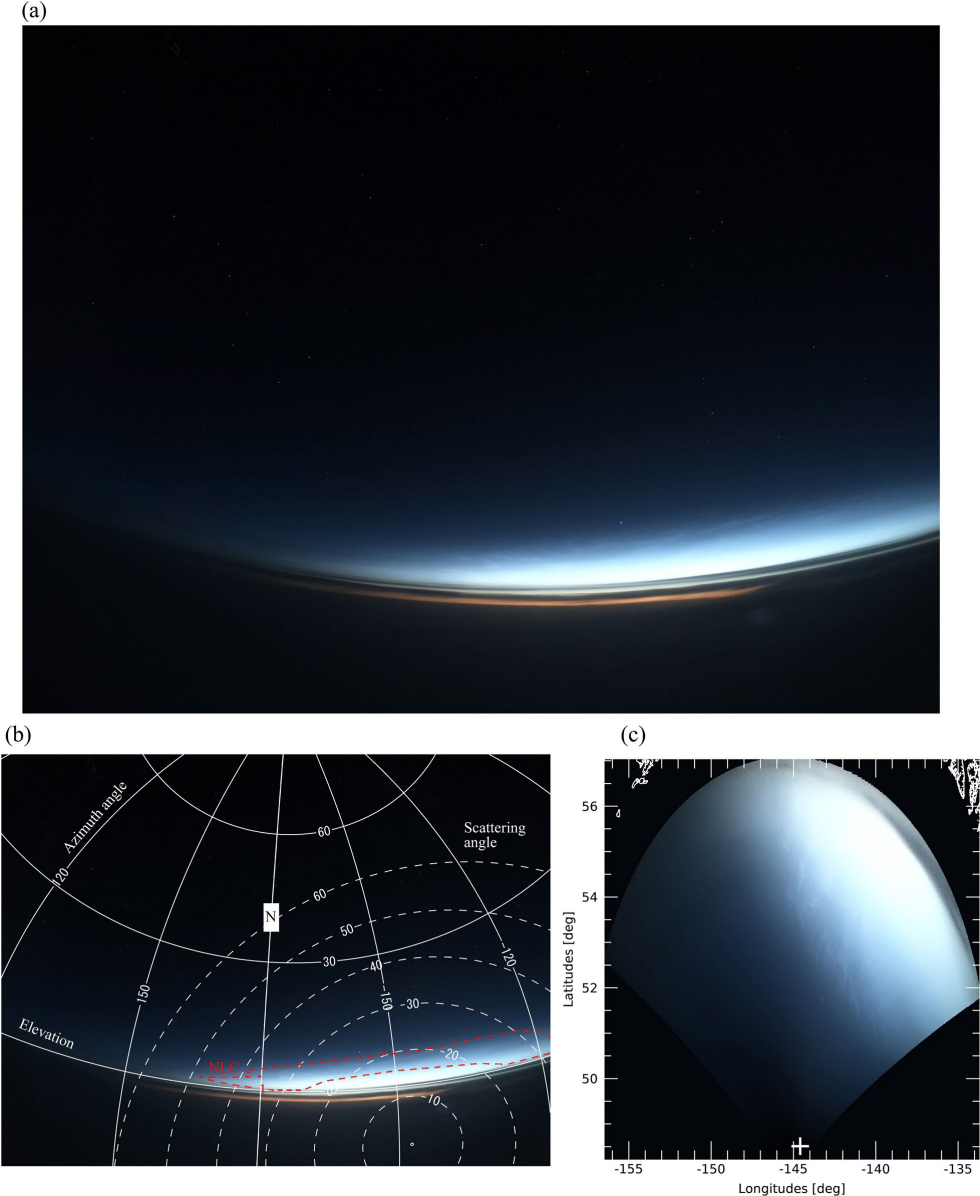
**a** Typical NLC image taken during flight NH105 at 12:45 UTC on Jul 8, 2020, over the northern Pacific Ocean. **b** Same image as (**a**), but with the horizontal coordinates embedded. The horizontal (vertical) lines show the elevation (azimuth) angle in 30° intervals. The notation “N” on the vertical line in the center of the image represents the geographical north. The area with NLC features is indicated by a red dotted line. The white dotted lines show the scattering angle. **c** Image projected onto geographical map by assuming an NLC altitude of 85 km. The plus symbol indicates the location of the jet during the observation

We then checked all taken sequential images for a potential presence of NLCs during each flight (see Table 1). The NLC visibility condition is defined as a solar elevation between -15 and -5° and an altitude of jets is above lower clouds. Actual routes of each flight were obtained from Flightradar24 (https://www.flightradar24.com/). The solar elevation angle during each observation was calculated to judge whether each image was taken under the NLC condition by combining a timecode of each image and the flight route log (time, latitude, longitude, and altitude). If an image was found to be taken under the possible NLC condition, then the image was carefully checked to determine whether it captures NLC features. The detection of NLCs in each image was completely made manually because NLCs have apparent features (bluish color and shining against a dark sky) in color images and are easily distinguished from lower clouds. Figure 1a shows a typical NLC image taken during flight NH105 at 12:45 UTC on Jul 8, 2019, over the northern Pacific Ocean. The solar elevation angle at this observation time was -12.3°, satisfying the NLC visibility condition. As shown by this example, many small-scale structures, modulation by gravity waves, are also seen in addition to the NLC features mentioned above. This is also a typical feature of an NLC image taken from the ground (Pautet et al. 2011).

The images with NLC features were then analyzed using the scheme described in Suzuki et al. (2015, 2016). This scheme involves deducing camera parameters (optical distortion coefficients) and fitting of a local horizontal coordinate system (elevation and azimuthal angles) to each pixel using reference star positions captured in the analyzed images. The simple distortion model considers distortion being proportionate to angular distance from the center of the image. The distortion model of the camera adopted in this study is the same as that described in Suzuki et al. (2018). Figure 1b is the same image shown in Fig. 1a but with the horizontal coordinates determined and embedded using this method. The horizontal and vertical curves in Fig. 1b show elevation and azimuth angles, respectively, in 30° intervals. The azimuth angle is set to zero at geographical south and increases toward the west-north-east direction (i.e., clockwise increment). The notation “N” on the curve in the center of the image represents geographical north. The area with NLC features appears at a low elevation area and is indicated by the red dotted line. The white dotted lines show the scattering angle (i.e., angle between a line connecting the observer and NLCs, and a line connecting NLCs and the Sun). The scattering angle is revisited in Sect. 4. Figure 1c shows the image projected onto a geographical map by assuming the typical NLC altitude of 85 km (Hansen et al. 1989). The latitudinal and longitudinal extents of the NLCs shown by this projection are from 51°N to 55°N and from 137°W to 146°W, respectively. The image area fairly closed to the horizontal line (elevation ~ 0) is saturated due to a strong background signal and thus unfortunately contains no information on the presence of NLCs. This situation is typical for all observations. Because the exposure time for each observation is determined automatically by the camera, this saturation problem frequently occurs when imaging a scene with a wide range of radiance (from a dark sky to a bright twilight sky). This problem limits the maximum range of observation. As shown in Fig. 1c, the effective range of the observation is about + 5° in latitude and at about + 500 km from the location of a jet if this effect is considered. In other words, NLCs observed at latitudes lower than 55°N means that the NLCs exist at latitudes lower than 60°N. Therefore, we regard NLC detection from latitudes lower than 55°N as a case of detection of mesospheric clouds at middle latitudes (< 60° N) by jets throughout this paper.

## Results

**Fig. 2:**
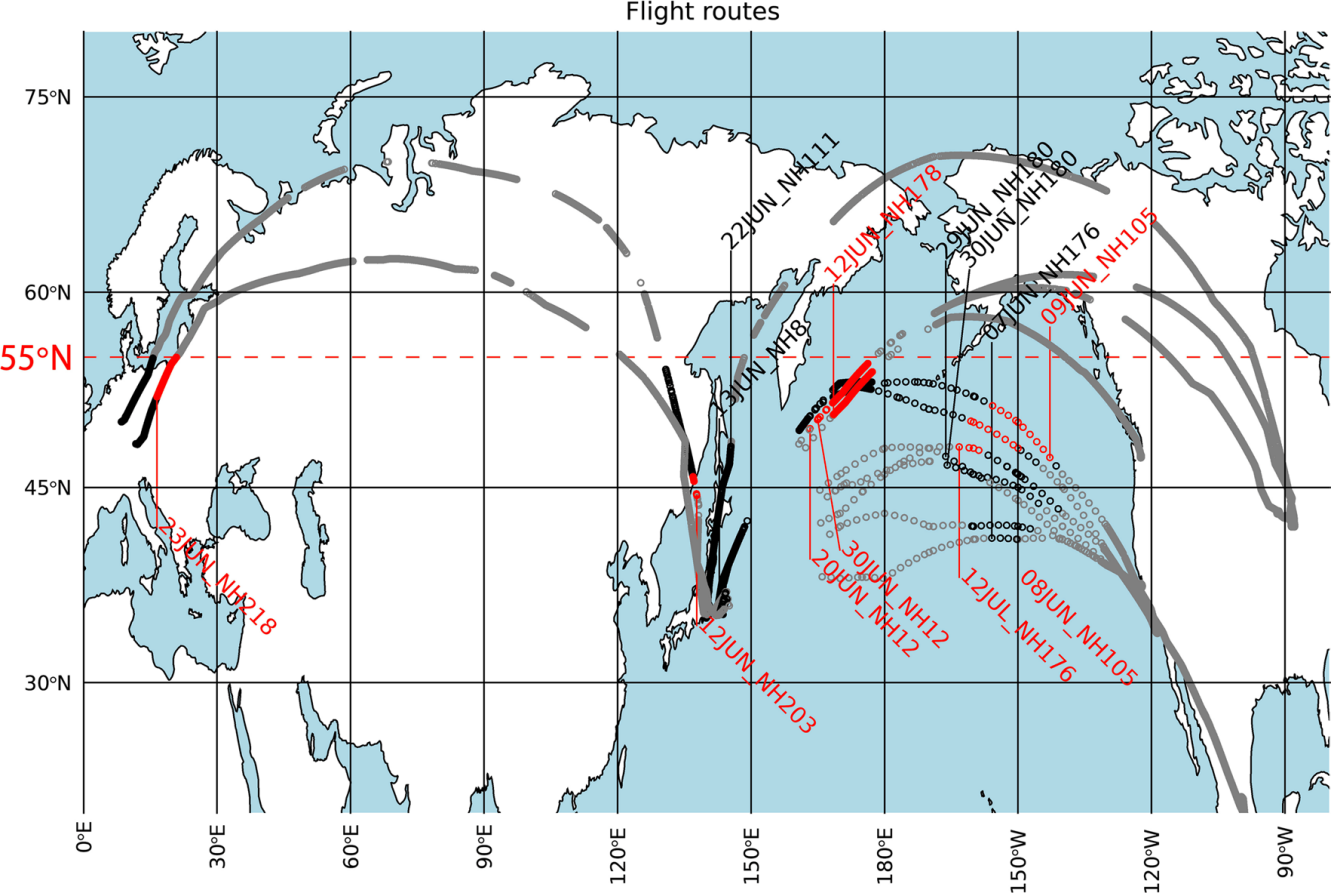
Flight routes and summary of the NLC observations conducted on 13 flights in the Northern Hemisphere during the summer 2019 (see Table 1). Gray and black circles on flight routes show the locations without and with a possibility of NLC detection, respectively. Red circles show locations from which NLCs were successfully detected

All results of the NLC observations in 2019 are summarized in Fig. 2. Actual flight routes for all 13 flights are taken from Flightradar24. The flight ID (e.g., NH203) and date of departure are also indicated for each flight. Gray circles in a flight route show the locations where the jet location did not satisfy the condition for NLC detection (see the “Method” section for the criteria). The black circles in a flight route show locations with a chance of NLC detection from the jet. The red circles show locations from which NLCs were detected. Intervals between these locations are not constant through each pass; they are sometimes intermittent and sometimes continuous due to irregular timing of flight reports from jets during cruising.

NLCs were detected from latitudes lower than 55°N on 8 of these 13 flights. Most detections were achieved over the Pacific Ocean. Because the Pacific Ocean contains few land areas with large populations, previous reports of NLCs from these areas are quite rare. Again note that locations with red circles plotted in Fig. 2 are not locations where NLCs existed but the locations from which NLCs were seen. The actual locations of NLCs observed from jets would be more poleward, as shown in Fig. 1c. This paper focuses on NLC detections from latitudes lower than 55° N to discuss the actual occurrence of NLCs in middle latitudes (< 60° N).

**Fig. 3:**
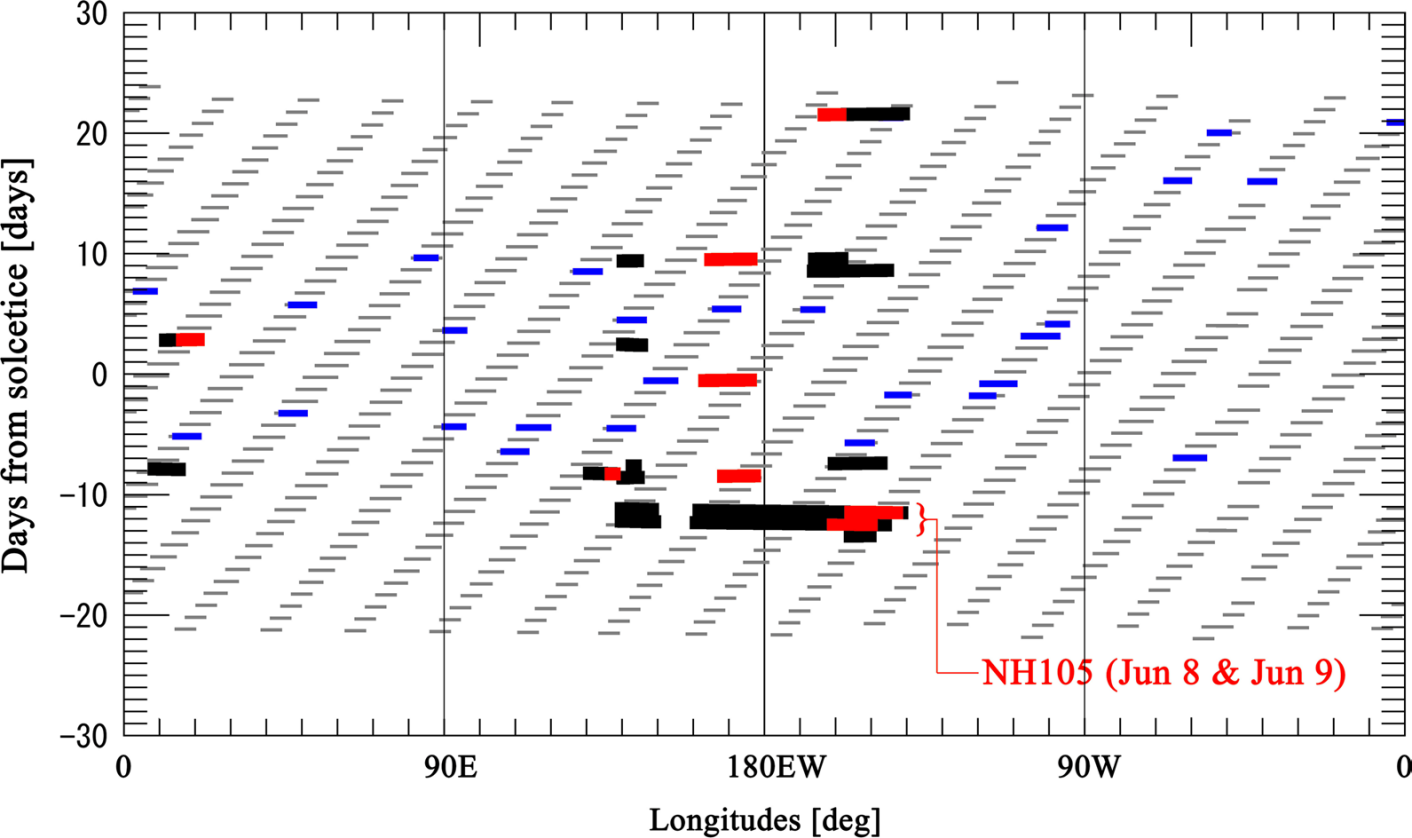
Temporal and longitudinal variation of NLCs detected by jets and the CIPS instrument onboard the AIM satellite. The vertical and horizontal axes represent days from north hemispheric summer solstice (DFS) in 2019 and longitudes, respectively. Red crosses and black plus symbols show the locations and times of NLC observations conducted by jets. As in Fig. 3, red crosses mean that NLCs were detected “from” these points, but plots are limited to latitudes lower than 55°N. The blue plus and gray plus symbols represent NLCs detected from space by the AIM/CIPS instrument. The horizontal width of grey lines roughly corresponds to the zonal distance of a foot print of CIPS camera in at latitude 55°N. All sampling points by AIM/CIPS between latitudes 50°N and 60°N are shown in this plot.

Figure 3 shows temporal and longitudinal distribution of sampling points of NLC observations by the jets and the CIPS instrument onboard the AIM satellite. The vertical and horizontal axes represent days from north hemispheric summer solstice (DFS) in 2019 and longitudes, respectively. Red and black symbols show the locations and times of NLC observations conducted by jets. As in Fig. 2, red crosses mean that NLCs were detected “from” these points, but plots are limited to latitudes lower than 55°N. The blue and gray symbols represent locations and times of PMC observations from space by the AIM/CIPS instrument. The horizontal width of gray lines roughly corresponds to the zonal distance of a foot print of CIPS camera at latitude 55°N. The version of data provided by the AIM/CIPS team is level 3c, version 05.20, revision 5. In this case, blue symbols mean that NLCs were detected “at” these points, but data are limited to latitudes between 50 and 60° N. Therefore, Fig. 3 is a combined result of the NLC presence in middle latitudes (< 60° north), confirmed by both the jets and AIM satellite in the Northern Hemisphere during the summer 2019. The observation coverage of test flights is shown in this figure. Some flights show remarkably wide longitude coverage of an NLC observation. In particular, the flight NH105 tested on Jul 8 and 9 (marked in Fig. 3) covers nearly 70 degrees width in longitude above middle of Pacific Ocean. Since all flights are daily scheduled, an NLC observation is possible with one day interval in this longitude range if the camera is installed on every jets. An expected distribution of sampling points in time and longitude of flight NH105 through the NLC season is shown and discussed in the next section.

It is also shown that in several cases, NLCs were detected only by jets, and AIM did not detect NLCs at the same time and location. For example, focusing on longitudes near 180°, NLC occurrence in the middle latitudes is only one detection between DFS-20 and DFS + 25, according to the AIM data. However, it increases to four events if both data sets are combined. This shows a difference in detection sensitivity for NLCs in middle latitudes between the jet and AIM satellite observations. We discuss this in more detail in the next section.

## Discussion

**Fig. 4:**
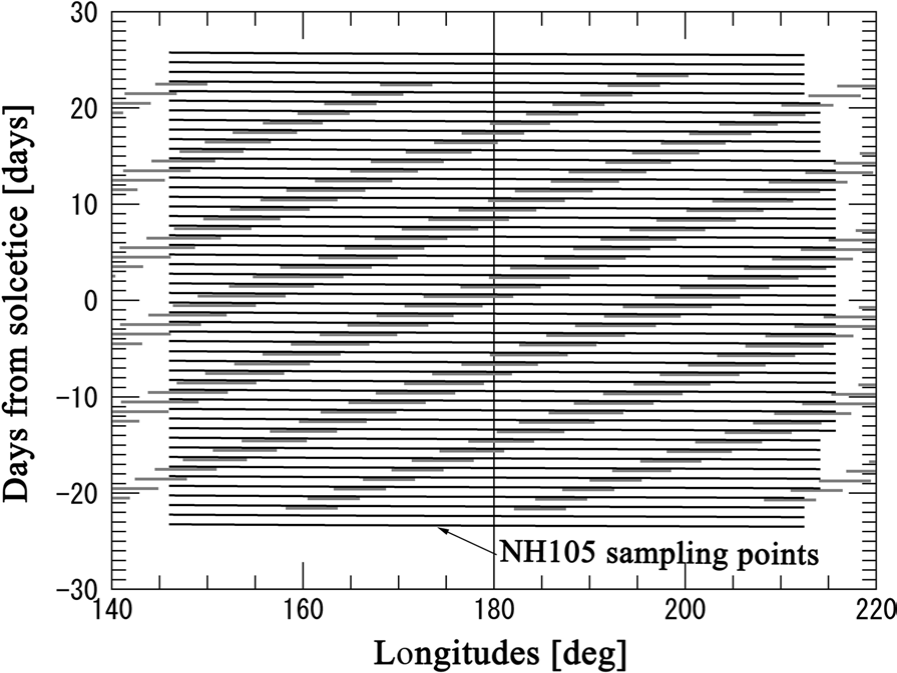
An expected distribution of sampling points in time and longitude of NH105 if the camera is installed on every flight through the NLC season. Possible sampling points by NH105 flights are shown by nearly horizontal lines

As shown in Fig. 3, a flight route of NH105 satisfies the criteria for an NLC observation (see the “Method” section for the criteria) with a long duration. Test observations with this flight have been conducted in two successive days (Jul 8 and Jul 9) and both show similar result. Since NH105 flights westward, a duration of twilight time becomes long. This allows NLC observation with NH105 to be possible in a wide longitude range. Figure 4 shows the expected distribution of sampling points in time and longitude of NH105 if the camera is installed in every flight through the NLC season. The flight route is assumed to be same as it on Jun 8. Variation of solar deceleration and right ascension during the period is considered to check the criteria for an NLC observation. Sampling points by AIM/CIPS instrument in latitudes between 50 and 60 degrees are also indicated by grey lines as well as Fig. 3. One can clearly see that there are significant spatial voids of 17–18 degrees between adjacent orbits of the AIM spacecraft at middle latitudes due to the spacecraft orbiting the Earth. These voids in the spatial midlatitude coverage produce gaps in PMCs observations by the CIPS instrument at middle latitudes as was mentioned in Dalin et al. (2020a). This fact is one of the causes that makes difficult to observe NLCs in the middle latitude region by a satellite in a near-polar orbit. On the other hand, expected sampling points based on the test observation by NH105 flight show much denser distribution in longitude between 145° E and 215° E. We also see several NLC observations as seen from jets (red marks in Fig. 3) located between AIM orbit trajectories (stripy distributed grey marks in Fig. 3), i.e., these NLC occurrences were missed by the CIPS cameras.

**Fig. 5:**
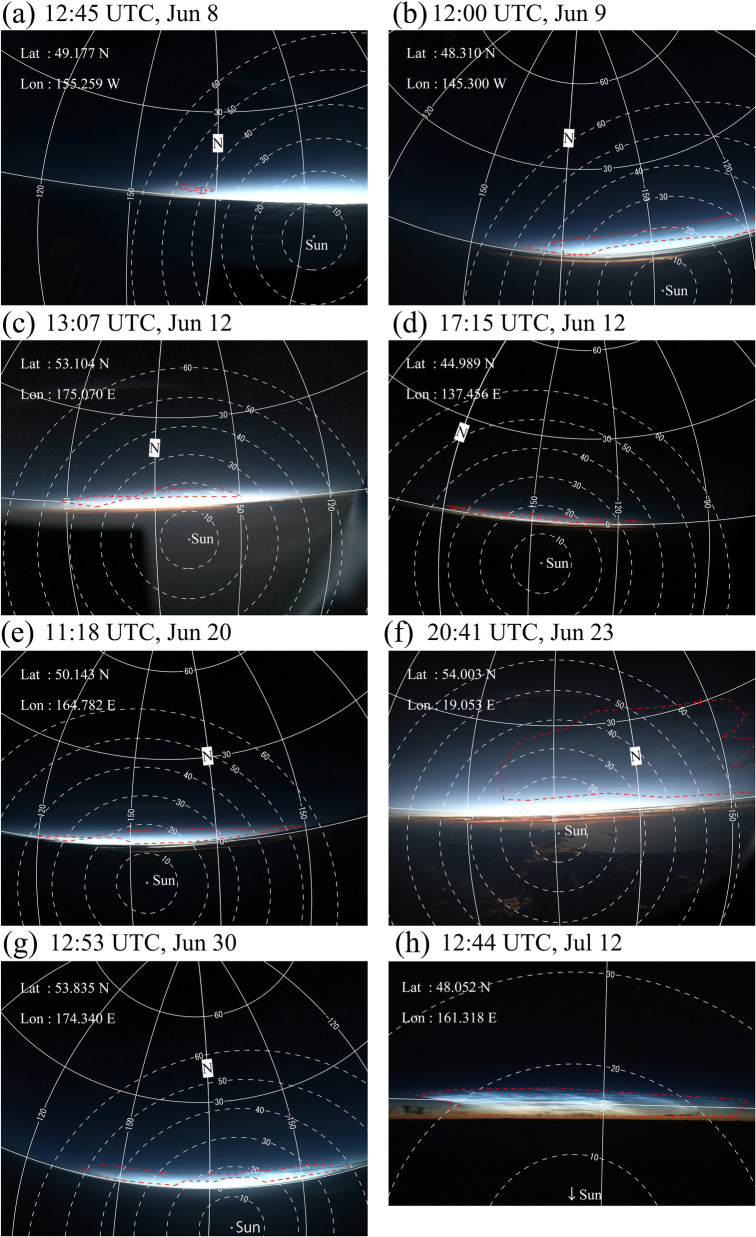
Selected images of NLCs taken by all eight flights that successfully observed NLCs from latitudes lower than 55°N. The precise location and other parameters of each observation are listed in Table 2. The meaning of auxiliary lines in each figure is same as in Fig. 1b

**Fig. 6:**
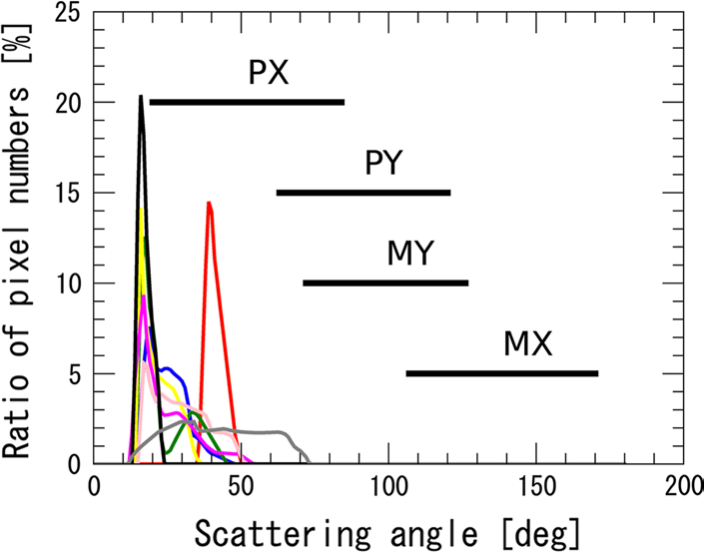
Distribution of scattering angles of pixels with NLC features. The distribution of the number of pixels is normalized by the total number of pixels with NLC features for all eight events (see Table 1 and Fig. 3), and each event is plotted in a different color. Typical ranges of scattering angles sampled by the four AIM/CIPS cameras (PX, MX, PY, and MY) when the SZA has the highest value (90°–95°) are also indicated (see Fig. 6 of Lumpe et al. 2013)

It is also shown that in several cases, NLCs were detected only by jets, and AIM did not detect NLCs at the same time and location. It is not straightforward to compare the detection limit of NLC signatures between jets and AIM/CIPS observations because of different wavelength, geometry, and sensitivities of sensors. However, the jet observation has an advantage in terms of an observation geometry. As shown in Fig. 1b, NLCs are typically seen poleward (or northward in the present case) at low elevations of the twilight sky from flight observers. Due to this geometry condition, the angle formed by the Sun, NLCs, and observer tends to be close to 180°. This means the scattering angle of NLCs is close to a forward-scattering condition (i.e., the scattering angle ≈ 0) when observations are conducted from the Earth’s surface. This tendency is shown in Fig. 5, which provides selected images of NLCs taken by all eight flights that successfully observed NLCs from latitudes lower than 55° N. The precise location of each observation is indicated in each panel and Table 2. The scattering angle at the observation time was calculated for all cases and shown by concentric dashed lines with an interval of 10°. The red dashed lines in each image show the image area in which NLC features are identified. Areas with saturated signals are omitted from the NLC area. The number of pixels with an NLC feature is sorted by the scattering angle and summarized as the histogram shown in Fig. 6. Each event is represented by a line with a different color. The scattering angles of pixels with NLC features are mostly distributed between 10° and 40° for all cases. In addition, clear peaks are also seen between 10° and 20° in most cases. These results show that NLC features observed from jets nearly satisfy forward-scattering conditions during observations. This works as an advantage for detection of NLCs because the phase function of scattering by particles measuring several tens of nanometers, which make up an NLC, shows strong scattering power around forward scattering (Bailey et al. 2009). In contrast, satellite observations are not conducted under such favorable conditions because they look at NLCs with a nearly nadir view from a polar orbit. The AIM/CIPS instrument consists of four nadir-viewing UV cameras that have a total field of view of 120° along the orbit and 80° across the orbit. These cameras are called the PX (+ X), MX (− X), PY (+ Y), and MY (− M) cameras after their pointing directions (Lumpe et al. 2013). A range of scattering angles sampled by AIM/CIPS varies depending on the solar zenith angle (SZA) at the time of observation. When the SZA is large, the observation has a small scattering angle. Typical ranges of scattering angle sampled by the four cameras when SZA has the highest value (90°–95°) during an operation are indicated in Fig. 6. It is noted that only the PX camera can cover the scattering angles that are sampled by the jets. Moreover, scattering angles between 10 and 20°, which are typical conditions for the jet observation, are almost out of range, even with the PX camera. Although an SZA angle of 90°–95° is the best condition for AIM/CIPS to detect NLCs (i.e., polar mesospheric clouds), it is generally difficult to observe NLCs under forward-scattering conditions, unlike a jet observation. Therefore, the jets observation has advantage in observation geometry by means of a scattering angle condition.

**Table 2:** A list of observation parameters for selected images shown in Fig. 5

Image ID (see Fig. 5)	Date and time (UTC)	Flight ID	Latitude (°)	Longitude (°)	Mean local time	Flight altitude (km)	Solar elevation angle (°)
(a)	12:45, Jun 8	NH105	49.177 N	155.259 W	2:24, Jun 9	9.4	− 11.167
(b)	12:00, Jun 9	NH105	48.310 N	145.300 W	2:19, Jun 9	9.8	− 12.298
(c)	13:07, Jun 12	NH178	53.104 N	175.070 E	0:47, Jun 13	12.5	− 13.061
(d)	17:15, Jun 12	NH203	44.989 N	137.456 E	2:25, Jun 13	9.8	− 14.346
(e)	11:18, Jun 20	NH12	50.143 N	164.782 E	22:17, Jun 20	10.1	− 12.955
(f)	20:41, Jun 23	NH218	54.003 N	19.053 E	21:57, Jun 23	11.3	− 7.952
(g)	12:53, Jun 30	NH12	53.835 N	174.340 E	0:30, Jul 1	9.8	− 12.817
(h)	12:44, Jul 12	NH176	48.052 N	161.318 W	1:59, Jul 12	10.4	− 15.614

## Conclusion

In June 2019, we started a project to monitor NLCs in middle latitudes from airplanes and balloons in addition to current ground-based imaging efforts (Dalin et al. 2020b). NLC observation from air-borne platforms has great advantage with respect to ground observation because the NLCs are not obstructed by lower clouds. Present work reports the initial results of observation of middle latitude NLCs from ANA jets conducted in the Northern Hemisphere during the summer 2019. NLC imaging observation was conducted on a trial basis with 13 flights during this season. NLCs were detected from middle latitudes (lower than 55° N) on 8 flights of 13 test flights. Temporal and longitudinal variations of the NLCs detected by jets and the CIPS instrument onboard the AIM satellite were compared. As a result, some ANA flights show remarkably wide longitude coverage of an NLC observation. For example, the NH105 flights westward from USA to Japan covers the nearly 70° longitude range above middle of Pacific Ocean since it satisfies the condition for the NLC observation with a long duration flight. Moreover, since all flights are daily scheduled, an NLC observation is possible with one day interval in wide longitude range if the camera is installed on every jets.

In several cases, NLCs were detected only by jets, and AIM did not detect NLCs at the same time and location. Though it is generally difficult to directly compare the detection sensitivity of these instruments, difference in the observation geometry is considered to be one of possible factor to explain this discrepancy. Observation geometries (i.e., scattering angle of NLC sampling) for all eight events were focused. It is found that observation of NLCs from jets possible has an advantage in detection sensitivity because imaging is performed under nearly forward-scattering conditions. This can be said also for ground-based imaging observation. However, observation from an airborne platform is free from obstruction by clouds in the lower atmosphere, strong tropospheric turbulence, light and aerosol pollutions. Thus, NLC observation from an elevation much higher than most tropospheric clouds has the potential to achieve continuous monitoring of faint signatures from NLCs in middle latitudes. Moreover, jets also allow NLCs to be observed not only over land but also over vast ocean areas. Based on the above facts, the advantages of an observation of midlatitude NLCs from jets are summarized as (1) wide longitudinal coverage compared to a satellite observation in one path, (2) no obscuration by lower clouds, (3) a favorable geometric condition for strong scattering signal from NLCs, and (4) possibility of observing NLCs over the oceans compared to ground-based measurements. On the other hand, a disadvantage is that an observation plan depends on the schedule of the airline jets which are determined by an airline company. In fact, observation plans in 2020 and 2021 have been unfortunately canceled because of a significant reduction of the number of international flights due to the global impact of COVID-19 pandemic.

The present work shows that regular observation using airline jets is a powerful tool to monitor the NLCs in midlatitude region with much denser sampling interval both in time and space than those of existing techniques (from the ground and space). A simple small camera can monitor NLCs in the middle latitude region covering wide longitude range if it is installed inside a cockpit of intercontinental regular flights.

## Data Availability

The NLC image data taken by jets used in this paper are available upon request to the corresponding author HS (suzuhide@meiji.ac.jp). The AIM-CIPS data are provided through http://lasp.colorado.edu/aim/index.php.

## Abbreviations

AIM: The Aeronomy of Ice in the Mesosphere
ANA: All Nippon Airways
CIPS: The cloud imaging and particle size
DFS: Days from summer solstice
FOV: Field of view
MSE: Mesosphere summer echo
NLC: Noctilucent cloud
NASA: National Aeronautics and Space Administration
PMC: Polar mesospheric cloud
SZA: Solar zenith angle
